# Towards the generation of gnotobiotic larvae as a tool to investigate the influence of the microbiome on the development of the amphibian immune system

**DOI:** 10.1098/rstb.2022.0125

**Published:** 2023-07-31

**Authors:** Abigail J. Miller, Jordan Gass, Myung Chul Jo, Lucas Bishop, Juli Petereit, Douglas C. Woodhams, Jamie Voyles

**Affiliations:** ^1^ Department of Biology, University of Nevada, Reno, NV 89557, USA; ^2^ Environmental Health and Safety, University of Nevada, Reno, NV 89557, USA; ^3^ Nevada Bioinformatics Center, University of Nevada, Reno, NV 89557, USA; ^4^ University of Massachusetts Boston, Boston, MA 02125, USA

**Keywords:** *Batrachochytrium dendrobatidis*, chytridiomycosis, ecoimmunology, infectious disease, immune priming, priority effects

## Abstract

The immune equilibrium model suggests that exposure to microbes during early life primes immune responses for pathogen exposure later in life. While recent studies using a range of gnotobiotic (germ-free) model organisms offer support for this theory, we currently lack a tractable model system for investigating the influence of the microbiome on immune system development. Here, we used an amphibian species (*Xenopus laevis*) to investigate the importance of the microbiome in larval development and susceptibility to infectious disease later in life. We found that experimental reductions of the microbiome during embryonic and larval stages effectively reduced microbial richness, diversity and altered community composition in tadpoles prior to metamorphosis. In addition, our antimicrobial treatments resulted in few negative effects on larval development, body condition, or survival to metamorphosis. However, contrary to our predictions, our antimicrobial treatments did not alter susceptibility to the lethal fungal pathogen *Batrachochytrium dendrobatidis* (*Bd*) in the adult life stage. While our treatments to reduce the microbiome during early development did not play a critical role in determining susceptibility to disease caused by *Bd* in *X. laevis*, they nevertheless indicate that developing a gnotobiotic amphibian model system may be highly useful for future immunological investigations.

This article is part of the theme issue ‘Amphibian immunity: stress, disease and ecoimmunology’.

## 1. Introduction

The immune system does not rest [1]. Rather, the equilibrium model of immunity suggests that it is perpetually responsive and dynamic, even under germ-free conditions or in the presence of symbionts [1–3]. This conceptual model further posits that early life conditions, especially exposure to microbes during development, prime immune responses for secondary exposures [4]. Accordingly, the timing and specificity of host–microbe interactions within early developmental windows may determine long-term immunological balance and therefore susceptibility to infectious microbes and disease development later in life [5–7].

The recent development of gnotobiotic (i.e. germ free) model organisms offers unparalleled opportunities to explore questions concerning host–microbe interactions and immune equilibrium [8]. For example, gnotobiotic organisms have helped to demonstrate the mechanisms of microbiome assemblage (e.g. via environmental and vertical transmission of microbes; [4,9]). Furthermore, new research shows that priority effects—the timing and the order in which microbes colonize a host—influence microbiota assembly [10,11] and may have important consequences for immunological development [12]. Yet, for all the promise that gnotobiology offers, our ability to conduct complex investigations into how microbial exposure modulates immune system development is somewhat hampered by the limitations of existing model systems [13]. For example, the most common gnotobiotic model organisms (e.g. mice, pigs) require some level of parental investment in offspring during development [14,15]. We lack effective methods to manipulate the microbiome during early life stages (decoupled from parental care) to fully understand microbial influence during critical developmental periods. As such, establishing additional model systems that allow researchers to strategically manipulate the historical contingency and exposure to microbiota during early development will help unravel the mechanisms that underpin host responses upon secondary exposure and, hence, resistance to infection and disease development in later stages of life [6,7].

Amphibians are a leading model system for immunological investigations [16–19]. Studies of amphibian functional microbiomes are revolutionizing our understanding of infectious disease systems [20–22]. Amphibians have highly sophisticated immune systems, including innate (non-specific) and adaptive (pathogen-specific) components [23,24]. In addition, a growing body of literature describes intensive investigations on the interplay among the amphibian immune system, the amphibian microbiome, and a wide variety of macro- and microparasites [6,23,25]. Furthermore, amphibians around the world are experiencing severe population declines and species extinctions due to infectious disease [26,27]. As such, research on amphibian immunity, and how it is affected by exposure to diverse microbes, provides a compelling and timely opportunity to investigate immune equilibrium hypotheses [21,25].

The emerging disease amphibian chytridiomycosis is a prime example of a host–pathogen system that can be used to investigate the role of the microbiome in immune priming and the mechanisms in disease dynamics [6,25]. This disease is caused by the fungal pathogen *Batrachochytrium dendrobatidis* (*Bd*) and has led to mass mortality events and disease-induced declines of amphibians around the world [26–29]. *Bd* colonizes the skin of amphibians, causing a variety of pathophysiological effects and mortality in a wide range of host species [30–32]. To date, no other pathogen is known to have such a ubiquitous effect on a broad range of host species and in so many different environments [27,33]. Thus, amphibians, and the disease chytridiomycosis, provide a useful system for investigating the respective roles of the microbiome and immune system development in a severe infectious disease system that has impacted hundreds of host species [27].

In this study, we conducted two experiments to investigate how reducing the microbiome during early development may alter development, body condition, and susceptibility to infectious disease. More specifically, we hypothesized that experimental reductions of microbiome richness and diversity would result in higher susceptibility in the amphibian host *Xenopus laevis* to the disease chytridiomycosis in later stages of life. To test this hypothesis, we conducted two experiments where we reduced the microbiome using two different approaches in tadpoles and reared them to metamorphosis in sterile and non-sterile conditions. In Experiment 1, we used one treatment with a single antimicrobial cocktail, administered at one time point, and also looked at the effect of sterilizing food to understand the relative contributions of microbes in larval food sources. In Experiment 2, we attempted to further reduce the microbiome by adding an additional antimicrobial cocktail (one that had additional antibacterial components) and administered the treatment at multiple timepoints. In addition, we allowed the tadpoles to undergo metamorphosis to measure the effects of antimicrobial treatments on body condition, larval development, and survival to metamorphosis. For the animals that completed metamorphosis in Experiment 2, we subsequently conducted an inoculation experiment to determine if the antimicrobial treatments altered susceptibility to *Bd* infection, disease development, and mortality.

## 2. Material and methods

In Experiment 1, we aimed to reduce the microbiome and understand the relative contributions of microbes in larval food sources. To do so, we reared tadpoles in one of four groups: antimicrobial treatment and sterile food (Group 1: AMX + SF), antimicrobial treatment and non-sterile food (Group 2: AMX + NSF), no antimicrobial treatment and sterile food (Group 3: No AMX + SF) and no antimicrobial treatment with non-sterile food (Group 4: No AMX + NSF).

In Experiment 2, we attempted to further reduce the microbiome by adding an additional antimicrobial cocktail (one with additional antibacterial components), which we administered multiple times. In total, Experiment 2 included five treatment groups: antimicrobial cocktail 1 with a single administration (Group 1: AMX1, 1x), antimicrobial cocktail 2 with a single administration (Group 2: AMX2, 1x), antimicrobial cocktail 2 administered at 2 time points (Group 3: AMX2, 2x), antimicrobial cocktail 2 administered at three time points (Group 4: AMX2, 3x), non-sterile tadpoles treated with a sham cocktail of sterile water and reared in non-sterile conditions (Group 5: No AMX). We allowed the tadpoles to undergo metamorphosis and conducted an inoculation experiment with *Bd* (electronic supplementary material, figure S1).

### (a) Sterile food and environments

To prepare for tadpole rearing in sterile conditions, we autoclaved stainless steel tanks (16.5 × 16 × 26.7 cm; Thunder Group, CA, USA) and placed them within a laminar flow biosafety cabinet. For the control animals, we placed identical tanks on a stainless-steel table adjacent to the cabinet, but in non-sterile laboratory space. Prior to the start of the experiment, we placed Petri dishes containing tryptic soy agar (TSA; 2.5 g Tryptic Soy Broth, 1.5 g agar, 100 ml water) in both locations to monitor for microbial growth inside and outside of the laminar-flow biosafety cabinet.

To prepare sterile food for tadpole rearing, we used *Xenopus* larvae tadpole powder (Carolina Biological; NC, USA), which we sterilized using gamma irradiation to feed the sterile treatment groups [34]. Gamma irradiation eliminates microbes without altering the nutritional content of food [35]. Prior to animal arrival in the laboratory, we conducted a preliminary experiment to determine the dose of gamma irradiation that would effectively sterilize the tadpole food (electronic supplementary material). We found that an irradiation range of 10–14 kGy effectively sterilized the tadpole food (electronic supplementary material, figure S2), and we used a dose of 15 kGy for Experiments 1 and 2.

### (b) Embryo arrival and antibiotic treatment

For both experiments, we ordered *Xenopus laevis* embryos from the Marine Biological Laboratory, National *Xenopus* Resource. The embryos arrived at approximately Nieuwkoop & Faber (NF) stage 16 [36]. Upon arrival, we randomly assigned the embryos to treatment groups and placed them in sterile 50 ml conical tubes. We then moved the embryos to be treated with an antimicrobial cocktail inside the laminar-flow biosafety cabinet and kept the non-sterile embryos outside the cabinet. We washed the embryos three times with 40 ml of sterile (i.e. autoclaved) water. After the third rinse with sterile water, we treated the embryos with a bath of one of two antimicrobial cocktails for 4.5 h.

For Experiment 1, we used an antimicrobial cocktail that has been tested in previous antimicrobial studies for amphibians [26]. Specifically, we added 500 µl of penicillin G:streptomycin, (10 000 units ml^−1^:10 mg ml^−1^), 50 µl of amphotericin B solution (250 µg ml^−1^) and 200 µl kanamycin sulfate (25 µg ml^−1^) to sterile deionized (DI) water to create a final total volume of 40 ml. We then mixed the solution well and filter-sterilized using 0.22 µm sterile syringe filter and luer lock syringe into a sterile 50 ml conical tube.

For Experiment 2, we tested a second antimicrobial cocktail that included additional antimicrobial components. We added 500 µl of penicillin G:streptomycin (10 000 units ml^−1^: 10 mg ml^−1^), 50 µl of amphotericin B solution (250 µg ml^−1^), 200 µl kanamycin sulfate (25 ug ml^−1^), 0.53 µl of sulfamethoxazole: trimethoprim (13.3 mg l^−1^: 2.67 mg l^−1^), and 1.2 mg enrofloxacin (final concentration 30 mg l^−1^) to a sterile 250 ml glass beaker and added sterile DI water to create a total volume of 40 ml. We mixed well and then filter-sterilized the solution using 0.22 µm sterile syringe filter and 50 ml luer lock syringe into a sterile 50 ml conical tube. We chose the additional antibiotics for their broad range of antimicrobial activity. Specifically, sulfamethoxazole–trimethoprim are known to target both Gram-positive and Gram-negative bacteria [37,38] and enrofloxacin is known to target several bacteria within the phylum *Proteobacteria* [39]. We therefore anticipated that the second antimicrobial cocktail would target a broader range of microbes.

For both experiments, we treated all embryos in the control (i.e. non-sterile) treatment groups outside of the laminar-flow biosafety cabinet with sterile water (containing no antimicrobials) for 4.5 h. After treatment with the antimicrobial or control solutions, we washed the embryos three times with 40 ml of sterile water. We then transferred the embryos to 75 cm^2^ tissue culture flasks containing 40 ml of fresh sterile water. After 5 days of embryo development (approx. NF stages 43–46; [36]), we transferred the embryos to randomly assigned tanks (i.e. autoclaved stainless-steel tanks) containing 500 ml sterile water.

### (c) Animal husbandry

Amphibian larval development can be influenced by tadpole densities as well as thermal conditions [40]. Therefore, we were careful to control for densities and thermal conditions for the sterile and non-sterile treatment groups. Following antibiotic treatment in Experiment 1, we separated animals by treatment group and housed tadpoles in four to five tanks per group. Following antibiotic treatment in Experiment 2, we separated animals by treatment group and housed tadpoles in two tanks per treatment group. We selected the number of tanks for each experiment based on the available space inside the biosafety cabinet that allowed us to maintain low tadpole densities (12–60 tadpoles per 1.5 l of water, depending on the experiment). To ensure that we maintained all groups in identical thermal conditions, we used ibutton loggers (Maxim Integrated Products, San Jose, CA, USA) to monitor temperature inside and outside the biosafety cabinet. We confirmed that the mean temperatures inside the biosafety cabinet (mean ± s.e.: 21.6°C ± 0.03) did not significantly differ from the mean temperatures outside the biosafety cabinet (mean ± s.e.: 21.6°C ± 0.02; *t*-test: *t*_8753.9_ = –0.27, *p* > 0.05).

We allowed water to dechlorinate for 24 h before sterilizing and transferring it into the tanks containing tadpoles. To verify sterility, we collected water samples from the tanks and added them to potato dextrose (39 g Potato Dextrose Agar, 1 l water) and TSA plates. We allowed the plates to incubate for a minimum of 24 h and visually inspected plates for microbial growth. We spot cleaned tanks as needed and added fresh water at minimum once a week. After week 5, we did a 50% water change twice weekly. After metamorphosis, we stopped using sterilized water for all treatment groups and continued to change frog water twice per week.

We sterilized tadpole food for antimicrobial treatment groups via gamma irradiation (as described above). We fed tadpoles *ad libitum* by adding fresh food to tadpole tanks once per week. We increased the feeding frequency and fed tadpoles twice and then three times per week once they were large enough to consume all the food in the tanks. After metamorphosis, we fed frogs aquatic pellets (Zoo Med; CA, USA) three times per week.

### (d) DNA extractions

On week five of tadpole development, we humanely euthanized *N* = 6 (Experiment 1) and *N* = 10 (Experiment 2) tadpoles per treatment group for 16S rRNA targeted amplicon microbiome sequencing analysis. We randomly selected tadpoles from each tank and weighed tadpoles to the nearest 0.1 g and measured snout-to-vent length (hereafter, SVL) to the nearest 0.1 mm to calculate body condition (mass/SVL; [41]). We euthanized the tadpoles using sterile MS-222 that was neutralized by adding sodium bicarbonate to pH 7.0 [42]. We then transferred the tadpoles to Powerbead Pro Tubes (Qiagen, Valencia, CA, USA). We homogenized the tadpoles using a tissue homogenizer (Mixer Mill MM 400; Retsch, Newtown, PA, USA) for 3 min each at 25 hz. We then extracted DNA from the homogenized samples using the QIAmp PowerFecal Pro DNA Kit (Qiagen, Valencia, CA, USA). We included *N* = 4 water blanks for Experiment 1 and *N* = 5 water blanks for Experiment 2, for which we followed the same protocol for extraction, substituting molecular grade water instead of a tadpole sample. We then shipped the samples on ice to the Idaho State University Molecular Research Core Facility for 16S rRNA sequencing. Additional methods for the 16S rRNA gene fragment amplification and library preparation are available in the electronic supplementary material.

### (e) Inoculation with *Batrachochytrium dendrobatidis*

In Experiment 2, we also conducted a *Bd* exposure experiment using the metamorphosed frogs to determine if experiencing development in a sterile environment altered susceptibility to chytridiomycosis. *Xenopus laevis* is regarded as resistant to chytridiomycosis [43,44], which made it an ideal species to determine if development in germ-free conditions altered susceptibility to infection, disease development, and mortality. As an additional advantage, we opportunistically conducted this exposure experiment at the same time as one with an additional host species, *Atelopus zeteki*, which is known to be highly susceptible to lethal chytridiomycosis [41,45]. Since we used the same *Bd* isolate, the groups of *A. zeteki* frogs could serve as a positive control for *Bd* pathogenicity and disease development (see electronic supplementary material).

Once all *Xenopus laevis* tadpoles completed metamorphosis (i.e. absorbed tails; [36]), we transferred them to individual plastic containers (19 cm × 11 cm × 14 cm) and added filtered tap water to a depth of 5–7 cm. We cleaned tanks, fed frogs aquatic frog pellets three times per week, and added fresh water twice per week. At the start of the inoculation experiment, we weighed all frogs to the nearest 0.1 g and measured SVL to the nearest 0.1 mm, which we used to calculate body condition (mass/SVL; [41]). We collected skin swab samples to test for *Bd* presence and infection intensity using standardized swabbing techniques [46].

We randomly assigned frogs to exposure and control groups and verified that individuals were distributed evenly across treatment groups (*N* = 8–10 frogs per group for antimicrobial-treated groups, *N* = 6–7 for non-sterile groups). We exposed the frogs to *Bd* or sham control solutions via three 24 h inoculum baths, each approximately 7 days apart. Specifically, we harvested *Bd* zoospores from isolate Rio Maria [47] by filtering liquid cultures. We chose this isolate because it was highly pathogenic in previous exposure experiments [41,47]. We determined *Bd* zoospore concentrations using a haemocytometer and adjusted the final concentrations of zoospores as needed by adding TGhL broth. We exposed the control group to sterilized TGhL media [48] diluted with the same volume, but without *Bd* zoospores [30,47].

For each exposure, we adjusted the volume of 20% Holtfretter's solution [48] and the size of the exposure containers to optimize the concentration of zoospores. For the first inoculation, we placed frogs in round plastic containers with a diameter of 11.5 cm and a height of 7.5 cm containing a bath of 68 ml of 20% Holtfretter's solution. We then added 2 ml of 1.44 × 10^6^ zoospores ml^−1^. For the second and third exposures, we placed frogs in round plastic containers with a diameter of 6.5 cm and a height of 2.5 cm containing a bath of 25 ml of 20% Holtfretter's solution and then added 1 ml of 1.40 × 10^6^ zoospores ml^−1^ and 1.70 × 10^6^ zoospores ml^−1^, respectively. Following the 24 h inoculation bath, we placed all frogs back in their original tanks. We continued to collect mass, SVL, and skin swab samples for diagnostic testing every two weeks until the termination of the experiment. Although we used similar bath inoculation methods for *Atelopus zeteki,* the exposure method and dose differed because *A. zeteki* is a terrestrial species whereas *X. laevis* is a fully aquatic species (see electronic supplementary material).

### (f) DNA extraction and qPCR amplification

We extracted DNA from our swabs using the DNeasy Blood and Tissue DNA Extraction Kit (Qiagen, Valencia, CA, USA). We then quantified *Bd* DNA on the swabs using real-time quantitative polymerase chain reaction (qPCR; [49]). We used an internal positive control (IPC; [50]) and a dilution set of plasmid standards (Pisces Molecular, Boulder, CO, USA) to quantify *Bd* load. We determined *Bd* load by using the cycle threshold (*C_t_*) value to calculate genomic equivalences. We also adjusted the *Bd* plasmid copy numbers by accounting for dilution during the extraction process [41].

### (g) Sequence data processing

We received fastq files from Idaho State University that were demultiplexed and had primers/adapters removed. For Experiment 1, we merged files in Mothur v. 46.1 and ran them through the University of Nevada, Reno BioX Core's standard pipeline for 16S V4 samples [51]. For Experiment 2, we analysed sequence data using Mothur v. 1.48.0 [51]. We assembled contigs and parsed based on unique barcodes attached to the 515F primer [52]. We discarded sequences without exact matches to the primer and barcodes used in the PCR amplification. We filtered sequences for quality with a 50-base sliding window, a minimum average quality score of 25, and eliminated those containing ambiguous bases, homopolymers (more than 8 bases), or having lengths > 300 bases. We aligned filtered sequences to the SILVA bacterial 16S rRNA database [53] that was truncated to contain the amplified V4 hypervariable region. We identified and removed chimeric sequences using the VSEARCH algorithm [54]. We classified the taxonomy of the remaining sequences using classify.seqs with default parameters but we removed sequences classified as eukaryotic, unknown, mitochondria or chloroplast. We rarefied samples to 50 000 sequences to scale all samples to the same magnitude. Quality-filtered sequences clustered into operational taxonomic units (OTUs) with a similarity threshold of at least 97%. We then imported the OTUs, taxonomy, subsampling, and phylogenetic information into R (v. 4.2.1) [55] for further downstream analyses. We evaluated the OTUs for frequency across samples and further filtered to retain only OTUs meeting a minimum count threshold.

### (h) Statistical analyses

We did statistical analyses using R v. 4.0.2 [50]. To understand differences in larval development, we compared the percentages of animals that successfully completed metamorphosis among all groups with 95% Clopper–Pearson exact confidence intervals (CI). We calculated body condition index at five weeks post embryo arrival (at the time of tadpole sacrifice for sequencing) by dividing tadpole mass by SVL. We then used ANOVAs with Tukey HSD *post hoc* tests, or non-parametric Kruskal–Wallis tests with *post hoc* Dunn tests, to compare body condition among our different treatment groups. To understand the differential, we used a Kaplan–Meier Survival analysis with a log rank test with a Benjamini–Hochberg *p*-value correction to look for pairwise comparisons among treatment groups [56].

To understand patterns in alpha diversity among groups, including OTU richness and Shannon diversity index (as there is typically a negative correlation between diversity and dominance indices, we also used Inverse Simpson), we used ANOVAs with Tukey HSD *post hoc* tests, or non-parametric Kruskal–Wallis tests with *post hoc* Dunn tests, to investigate differences in alpha diversity measures among our treatment groups. To search for important differences in the microbial communities among treatment groups, we used a Wilcox test to compare the number of genera that could be attributed to treatments (antibiotic application and food sterility). We used an adjusted (false discovery rate (FDR) correction) *p*-value of 0.05.

We also used a permutational multivariate analysis of variance using Bray–Curtis distance matrix [57] to compare the microbial community structure and determine the variance among samples explained by treatment. We used ordinations to visualize the treatment separation. To further investigate the diversity across experimental groups at a phyla level, we calculated group comparisons within each phylum and used Kruskal–Wallis tests to identify statistically significant differences across the distributions. We then used pairwise comparisons using Wilcoxon rank sum test to identify which treatment groups differed significantly within particular phyla.

For the *Bd* inoculation experiment, we calculated the change in body condition by subtracting body condition during the intitial week of the *Bd* exposure from body condition during the final week of the exposure. We used an ANOVA with Tukey HSD *post hoc* tests to compare body condition among control and the antimicrobial treatment groups. To understand how body condition changed over time, we ran a mixed-effects linear model with a Gaussian distribution to compare the body condition of all frogs exposed to *Bd* in each treatment group over the course of infection with an interaction between experimental day and treatment group and using individual frog as a random effect (package: ‘lme4’ [58]; package: ‘lmerTest’ [59]; package: ‘DHARMa’ [60]).

For the *Bd* loads for the groups of frogs exposed to *Bd* over the course of the exposure experiment, we used a mixed-effects linear model with a Gaussian distribution. We calculated pathogen load by log-transforming the genomic equivalents of *Bd* load from the qPCR data. We then used treatment group as a fixed effect, individual frog as a random effect, and included an interaction between experimental day and treatment group (package: ‘lme4,’ [58]; package: ‘lmerTest,’ [59]; package: ‘DHARMa,’ [60]).

## 3. Results

### (a) Microbial richness and diversity

For both experiments, we included water (blank) control samples to check for contamination in the sample preparation. For Experiment 1, there were 26 total OTUs across the water samples, and none were shared across all water samples. Additionally, there was no overlap of any OTUs with the tadpole samples in any of our treatment groups, indicating no contamination during the tadpole sample processing.

**Figure 1.**
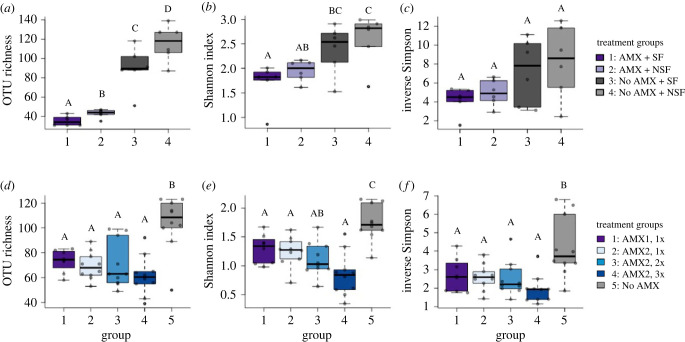
Microbial richness of groups of *Xenopus laevis* larvae treated with antimicrobial cocktail or sham control solutions and reared in sterile and non-sterile conditions. (*a,d*) The number of unique operational taxonomic units (OTUs, a common measure for richness) in tadpoles following five weeks of development in sterile and non-sterile conditions. (*b,e*) Shannon diversity index values, which measure richness and evenness of OTUs in a community, for groups of *X. laevis* tadpoles following five weeks of development in sterile and non-sterile conditions. (*c,f*). Inverse Simpson diversity values, which are an index of dominance of OTUs, for groups of *X. laevis* tadpoles following five weeks of development in sterile and non-sterile conditions. Box and whisker plots show median values with upper and lower quartiles and maximum and minimum values.

In Experiment 1, our antimicrobial treatments reduced microbial richness, diversity, and evenness for tadpoles reared with sterile and non-sterile food. We found differences in OTU richness among our treatment groups (Kruskal–Wallis: $\chi_{3}^{2}=19.36,$ *p* < 0.01). Specifically, we found that there was an incremental reduction in OTU richness in larvae in the antimicrobial treatment groups (mean ± s.e.: Group 1: AMX + SF: 35.3 ± 2.09; Group 2: AMX + NSF: 43 ± 1.77; figure 1*a*) compared to the no antimicrobial treatment groups (Group 3: No AMX + SF: 89.7 ± 9.05; Group 4: No AMX + NSF: 115.8 ± 7.66; figure 1*a*). We ran a *post hoc* Dunn test to look at pairwise comparisons of OTU richness and found differences among all treatment groups (Dunn test: all groups *p* < 0.01).

In addition, the Shannon diversity index, which is a composite measure of richness and evenness in a community, also differed among treatment groups (Kruskal–Wallis: $\chi_{3}^{2}=9.19,$ *p* = 0.03; figure 1*b*), with a *post hoc* Dunn test showing different Shannon diversity values between Group 2: AMX + NSF and Group 4: no AMX + NSF (Dunn test: *p* = 0.04; figure 1*b*), between Group 1: AMX + SF and Group 4: no AMX + NSF (Dunn test: *p* < 0.01; figure 1*b*), and between Group 1: AMX + SF and Group 3: No AMX + SF groups (Dunn test: *p* = 0.04; figure 1*b*). Our analysis of the inverse Simpson Index, which is a dominance index that accounts for the proportion of taxonomic units in a sample, showed no difference among treatment groups (ANOVA: *F*_3,20_ = 2.84, *p* = 0.064; figure 1*c*).

In Experiment 2, the tadpoles in the antimicrobial treatment groups had reduced microbial richness, diversity and evenness for all tadpoles treated with antimicrobials. Using an ANOVA with a *post* *hoc* Tukey HSD, we found significant differences in OTU richness between each antimicrobial-treated group and Group 5: No AMX (ANOVA: *F*_4,43_ = 10.03, *p* < 0.01; Tukey HSD, all groups *p* < 0.01; figure 1*d*). We observed a trend of decreasing OTUs with an increasing number of antimicrobial treatments (mean ± s.e.: Group 1: AMX1, 1x: 73.5 ± 3.16; Group 2: AMX2, 1x: 69.3 ± 3.46, Group 3: AMX2, 2x: 70.8 ± 6.01; Group 4: AMX2, 3x: 61.4 ± 4.92; figure 1*d*), although these differences among groups were not significant.

We found a very similar pattern with the Shannon diversity index. Specifically, the Shannon diversity was reduced in all antimicrobial-treated groups compared to the group that did not receive antimicrobial treatment (ANOVA: *F*_4,43_ = 12.08, *p* < 0.01; Tukey HSD, Group 1: AMX1, 1x compared to Group 5: no AMX *p* = 0.02, all other groups *p* < 0.01; figure 1*e*). Similar to OTU richness, we observed a decrease in diversity with higher numbers of antimicrobial treatments (figure 1*e*). We found significant differences between Group 1: AMX1, 1x (Tukey HSD, *p* = 0.02; figure 1*e*) compared to Group 4: AMX2, 3x. We also found significant differences between Group 2: AMX2, 1x (Tukey HSD, *p* = 0.03; figure 1*e*) and Group 4: AMX2, 3x. We used an ANOVA with a *post hoc* Tukey HSD test to look at pairwise differences in Inverse Simpson diversity and found a reduction in all antimicrobial-treated groups compared to Group 5: no AMX (ANOVA: *F*_4,43_ = 6.65, *p* < 0.01; Tukey HSD, Group 1: AMX1, 1x compared to Group 5: no AMX *p* = 0.03, all other all groups *p* < 0.01; figure 1*f*). In addition, we observed that the measures of richness and diversity tended to be decreased with increased frequency of antibiotics treatments (figure 1*d–f*).

### (b) Microbial community composition

**Figure 2.**
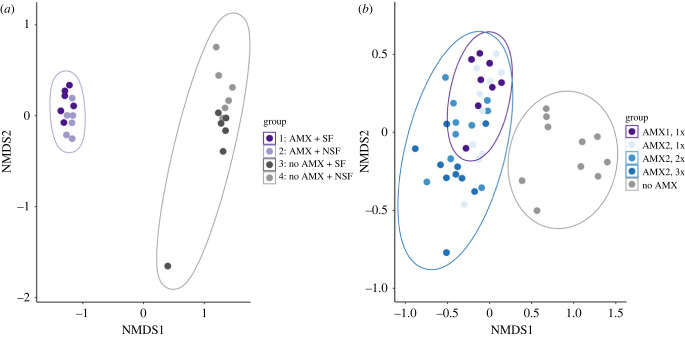
Non-metric multidimensional scaling (NMDS) ordination of groups of tadpoles treated with antimicrobial cocktail or sham control solutions and reared in sterile and non-sterile conditions. (*a*) Groups of tadpoles after antimicrobial (AMX; purple points and ellipse) or sham control treatments (no AMX; grey points and ellipse) and reared with sterile food (darker shade of purple and grey points) or non-sterile food (lighter shades of purple and grey points). (*b*) NMDS ordination of treatment groups of tadpoles after a single treatment of antimicrobial 1 (AMX1; purple points and ellipse) or sham control treatments (no AMX; grey points and ellipse) or antimicrobial 2 (AMX2; blue points and ellipses) that were administered once (light blue points), twice (medium blue points) or three times (dark blue points).

Although there was some variation in microbial community composition among all the treatment groups, we found considerable differences between the antimicrobial-treated and non-sterile groups of tadpoles. For Experiment 1, non-metric multidimensional scaling (NMDS) plots of the microbial communities in individual tadpoles revealed two clusters because of antimicrobial and non-sterile treatments (figure 2*a*). Furthermore, we found that antibiotic treatment (i.e. maintenance in sterile conditions) explained 39% of the community structure (PERMANOVA, adonis test, *p* < 0.01; figure 2*a*), whereas only a small percentage (5%) of the variance was explained by food sterility. We also used pairwise tests to understand which microbial genera differed among groups and whether differences could be attributed to antibiotic treatments or food sterility. We found that most of the significant differences in relative abundance of different microbial genera were due to antibiotic treatments and fewer due to food sterility (electronic supplementary material, table S1).

**Figure 3.**
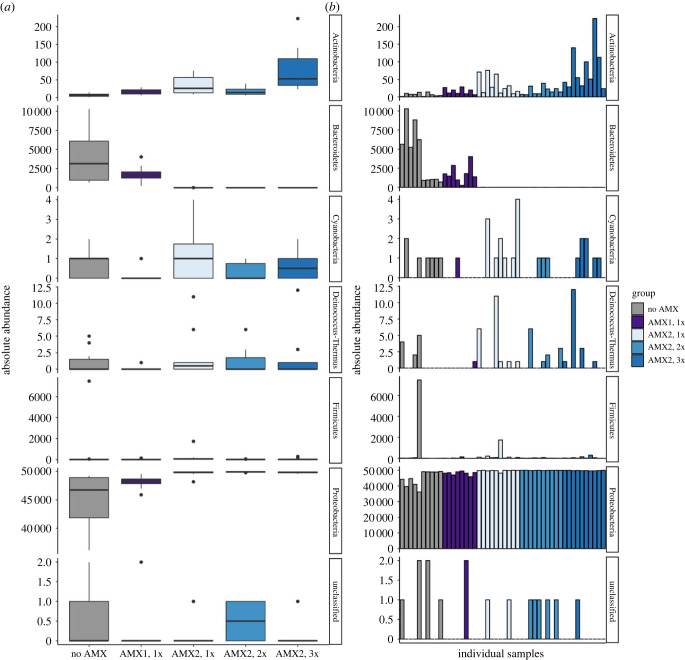
Rarefied total OTU abundances of microbes from multiple phyla in *Xenopus laevis* larvae treated with antimicrobial cocktail or sham control solutions and reared in sterile and non-sterile conditions. (*a*) Group mean and (*b*) individual absolute abundances in seven microbial phyla.

In Experiment 2, we found considerable differences between the groups that received antimicrobial treatments compared to the group that did not (Group 5). The NMDS plots of the microbial communities in individual tadpoles separated into two clusters that corresponded with antimicrobial treatments (both cocktails 1 and 2) and the non-antimicrobial (i.e. non-sterile treatment; figure 2*b*). We found that antimicrobial treatment (i.e. maintenance in sterile conditions) explained 37% of the community structure (PERMANOVA, adonis test, *p* < 0.001; figure 2*b*). In addition, we observed that the rarefied total OTU abundances of microbes in different genera differed by antimicrobial treatments. For example, one of the phyla that showed statistically significant differences (*p* < 0.001) was Bacteroidetes (figure 3*a,b*). These bacteria were depleted when tadpoles were treated with the second antimicrobial cocktail (AMX 2; figure 3).

### (c) Development, body condition and survival

**Figure 4.**
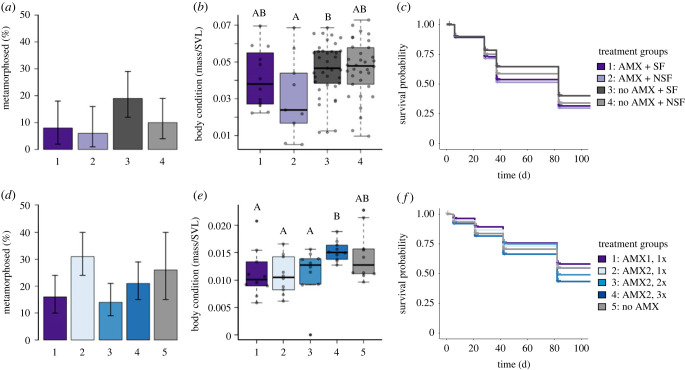
Development, body condition and survival in groups of *Xenopus laevis* treated with antimicrobial cocktail or sham control solutions and reared in sterile and non-sterile conditions. (*a,d*) Percentage of froglets (total number of frogs/initial number of embryos; not including tadpoles used for 16 s sequencing) of *Xenopus laevis* that successfully completed metamorphosis (% with 95% Clopper–Pearson confidence intervals). (*b,e*) Body condition of tadpoles taken five weeks after embryo arrival (calculated as mass/snout-to-vent length). (*c,f*) Survivorship of *X. laevis* tadpoles during development. Box and whisker plots show median values with upper and lower quartiles and maximum and minimum values.

In Experiment 1, we used the emergence of limb buds as a proxy for the duration of the larval period. We found no differences in the time to limb bud development among the treatment groups (ANOVA: *F*_3,15_ = 0.373, *p* > 0.05). In addition, we calculated the percentage of animals that reached metamorphosis among our treatment groups (excluding the tadpoles euthanized for sequencing). We found that a higher percentage of tadpoles in Group 3: no AMX + SF (19%, with 95% CI; figure 4*a*) completed metamorphosis. By contrast, relatively lower percentages of tadpoles completed metamorphosis in Group 1: AMX + SF (8%), Group 2: AMX + NSF (6%), and Group 4: no AMX + NSF (10%; with 95% CI for each group; figure 4*a*). In addition, we found that there was a slight difference in body condition at five weeks after embryo arrival (ANOVA: *F*_3,88_ = 2.724*, p* = 0.049; figure 4*b*). A Tukey HSD *post hoc* analysis revealed that the difference was between Group 2: AMX + NSF and Group 3: no AMX + SF (Tukey HSD, *p* = 0.047; figure 4*b*).

Our survival rates for *X. laevis* embryos were similar to non-experimental *X. laevis* embryos in previous studies [61,62]. In Experiment 1, survival rates differed among treatment groups, with greater survival in the groups that had no antimicrobial treatments (Group 3: no AMX + SF and Group 4: no AMX + NSF compared to Group 1: AMX + SF and Group 2: AMX + NSF; Kaplan–Meier, *χ*^2^ = 12.2, d.f. = 3, *p* < 0.01; figure 4*c*). There were significant pairwise differences between Group 1: AMX + SF and Group 3: no AMX + SF (log-rank test: *p* = 0.03), and Group 2: AMX + NSF and Group 3: no AMX + SF (log-rank test: *p* < 0.01). There were no significant differences in survival between Group 1: AMX + SF and Group 2: AMX + NSF (log-rank test: *p* > 0.05) or between Group 3: no AMX + SF and Group 4: no AMX + NSF (log-rank test: *p* > 0.05).

In Experiment 2, we found that the greatest percentage of tadpoles completed metamorphosis in Group 2: AMX2,2x (31%), followed by Group 5: no AMX (26%; figure 4*d*). By contrast, smaller percentages of tadpoles completed metamorphosis in Group 1: AMX1, 1x (16%), Group 3: AMX2, 2x (14%) and Group 4: AMX2, 3x (21%). We found differences in body condition at five weeks after arrival among the antimicrobial-treated groups (Kruskal–Wallis: $\chi_{4}^{2}=12.41,$ *p* = 0.01), with a *post hoc* Dunn test showing the differences were between Group 4: AMX 2, 3x and all other groups (Dunn test: Group 1: AMX 1, 1x, *p* < 0.01; Group 2: AMX 2, 1x, *p* < 0.01; Group 3: AMX 2, 2x, *p* = 0.03) except Group 5: No AMX (Dunn test: *p* > 0.05; figure 4*e*).

In Experiment 2, survival rates differed among treatment groups (Kaplan–Meier, *χ*^2^ = 43.8, d.f. = 4, *p* < 0.01; figure 4*f*). Group 1: AMX1, 1x survived significantly longer than Group 3: AMX2, 2x (log-rank test: *p* < 0.01), and Group 4: AMX2, 3x (log-rank test: *p* < 0.01). We found higher survival in Group 2: AMX2, 1x compared to Group 3: AMX2, 3x (log-rank test: *p* = 0.01) and Group 4: AMX2, 3x (log-rank test: *p* < 0.01). Lastly, Group 5: no AMX survived significantly longer compared to Group 4: AMX2, 3x (log-rank test: *p* < 0.01).

### (d) Inoculation with *Batrachochytrium dendrobatidis*

**Figure 5.**
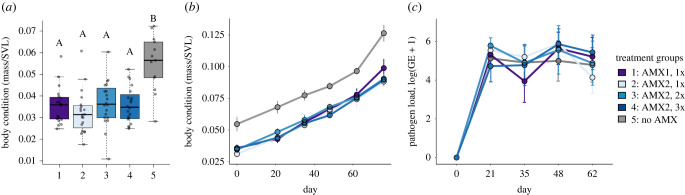
Body condition after metamorphosis, change in body condition over time in an inoculation experiment, and intensity of infection (pathogen load) after exposure to *Batrachochytrium dendrobatidis* (*Bd*). (*a*) Body condition of frogs taken after rearing in sterile and non-sterile conditions and three weeks after metamorphosis (calculated as mass/snout-to-vent length). (*b*) Body condition for all groups over time in weeks following *Bd* exposure (dots indicate mean intensity of infection as determined with quantitative PCR. Error bars indicate standard error of the mean). (*c*) Intensity of infection (pathogen load) after exposure for all groups throughout the *Bd* infection. Pathogen load was calculated as log(genomic equivalents (GE) + 1). Error bars indicate standard error of the mean.

Although body condition did not significantly differ between antimicrobial-treated groups and Group 5: no AMX prior to metamorphosis (figure 4*e*), there were differences after metamorphosis was complete (figure 5*a*). Specifically, at the start of our inoculation experiment (approximately three weeks after metamorphosis), we found significant differences in body condition among our treatment groups (ANOVA: *F*_4,88_ =11.98, *p* < 0.01; figure 5*a*). Using a Tukey-HSD *post hoc* to look at pairwise differences among groups, we determined that the difference was between all antimicrobial-treated groups and Group 5: no AMX (Tukey HSD, all groups *p* < 0.01; figure 5*a*). There were no differences among any of the antimicrobial treatment groups (Tukey-HSD, *p* > 0.05; figure 5*a*). Body condition changed over the course of the inoculation experimental day for all groups exposed to *Bd* (linear mixed model (LMM), *F* = 1555.9, *p* < 0.01; figure 5*b*). We also found significant interactions between experimental day and treatment group for Group 3: AMX2, 2x (LMM, *F* = 2.59, *p* = 0.04; figure 5*b*) and Group 4: AMX2, 3x (LMM, *F* = 2.59, *p* = 0.04; figure 5*b*). However, we found no differences in change in body condition among treatment groups (ANOVA: *F*_4,37_ = 1.87, *p* = 0.137; electronic supplementary material, figure S3).

For the *Xenopus laevis* frogs that were exposed to *Bd*, we found that the prevalence was 98% (all but 1 exposed frogs became infected). The infection intensities (i.e. *Bd* loads) increased over time for all treatment groups (LMM, *F* = 46.9, *p* < 0.01; figure 5*c*). We found no differences in infection intensity among our treatment groups (LMM, *F* = 0.07, *p* > 0.05), and no significant interactions between group and time among all treatment groups (LMM, *F* = 0.49, *p* > 0.05; figure 5*c*). We saw no clinical signs of disease or mortality in any treatment group of *X. laevis* frogs. In comparison, we found that all the exposed *A. zeteki* frogs became infected with *Bd*, exhibited clinical signs of severe chytridiomycosis [30] and died within 66 days following exposure.

## 4. Discussion

Recent research using a range of gnotobiotic model organisms suggests how historical contingency and priority effects (the order of microbial colonization) influence microbiome assembly, immune development, and health outcomes later in life. In germ-free mice, for example, the sequence of colonization by gut bacteria impacts the microbial community assembly, suggesting that variation in timing of exposure to microbiota may determine early microbial colonization events [11,63]. However, to date we have a limited number of model organisms to use for investigating questions concerning microbial exposure in early developmental windows [12].

In this study, we used two experimental approaches to reduce the richness of the microbiome in developing larvae of the model amphibian, the African clawed frog (*Xenopus laevis*). We found that our experimental manipulations using antimicrobial treatments (two different antimicrobial cocktails), sterile environments (i.e. autoclaved water), and sterile, gamma-irradiated food successfully reduced the richness of the microbiome in tadpoles with relatively few negative effects on body condition. We found that, overall, measures of development during the larval period, survival to metamorphosis, and body condition at the tadpole stage were comparable across all groups in both experiments. In addition, while body condition (e.g. body size) was lower following antibiotic treatment in post-metamorphic frogs, we did not observe higher susceptibility to the lethal pathogen *Batrachochytrium dendrobatidis* (*Bd*) as we had predicted. Taken together, these results suggest that exposure to antibiotics at this early stage of *X. laevis* development (NF stage 16 [36]) did not alter susceptibility to chytridiomycosis despite the experimental reduction of microbiome diversity and composition. Nevertheless, these findings demonstrate the utility of developing gnotobiotic tadpoles for future research and provides a novel model system for investigating microbiome assemblage, priority effects, and the impact of the microbiome on immune system development.

Antimicrobial treatments using two different cocktails of antibiotic and antifungal solutions successfully reduced the alpha diversity and altered the community composition of the microbiome in developing tadpoles. In Experiment 1, we found that antimicrobial treatments were more influential than food sterility at reducing microbial richness and diversity. In addition, in Experiment 2, we found that there were few significant differences among the groups that were treated with two different antimicrobial treatment cocktails. However, we observed striking trends of declining microbiome richness and diversity in tadpoles that experienced a higher number of administrations (three successive treatments) of the second antimicrobial cocktail, which included additional antibiotics (i.e. sulfamethoxazole, trimethoprim, enrofloxacin). In addition, we anticipated the second antimicrobial treatment would target more bacteria within the phylum Proteobacteria, but instead we observed that the second treatment eliminated the contribution of bacteria in the phylum Bacteriodetes (figure 3). Previous studies have made similar attempts to produce gnotobiotic amphibians (e.g. with Northern leopard frogs, *Rana pipiens*; [64]), but the investigators did not use irradiated food sources and observed high levels of mortality. Our findings suggest that additional manipulations of antimicrobial treatment during the development could eventually produce a truly axenic (i.e. gnotobiotic or germ-free) amphibian model system.

In both experiments, we found that reducing microbial richness and diversity had relatively few detrimental effects on tadpole body condition, successful development through metamorphosis during the larval period, and survival to the adult life stage. While there were some differences among groups, there were few consistencies across these experimental parameters that would suggest considerable negative effects of antimicrobial treatments. For example, in Experiment 2, Group 5: no AMX had higher body condition relative to the antimicrobial treatment groups post-metamorphosis. However, we noted that body condition of the frogs in these groups consistently improved over the course of the experiment (figure 5*b*). In fact, body condition improved equally among groups such that there were no differences in the change in body condition from the first timepoint to the last time point (electronic supplementary material, figure S3), irrespective of *Bd* infection. These findings are particularly encouraging for future research; since reduced body condition and overall health can be conflating factors in gnotobiology [65], developing a model organism that allows for microbiome manipulation with relatively few deleterious effects will be highly useful for a wide range of investigations.

We predicted that a significant reduction in microbiome richness and diversity during development would alter susceptibility to chytridiomycosis [6,66]. Our results did not support our hypothesis and instead indicated that our antimicrobial treatments did not affect infection intensity, disease development, or mortality in *X. laevis*. In contrast, the highly susceptible species *A. zeteki* (which we used as a positive control for *Bd* infection) exhibited high levels of disease and mortality with a lower *Bd* dose. This result provided evidence of the high pathogenicity of *Bd* used in this experiment, even though we observed no clinical signs of disease or mortality in any of the treatment groups of *X. laevis*.

Several previous studies have reported that the amphibian host species *X. laevis* is highly resistant to lethal effects of *Bd* infection [43,44]. In addition, a wide range of studies focused on understanding the functional role of the amphibian cutaneous microbiome have indicated that microbial richness confers resistance to *Bd* infection and reductions in the negative effects of chytridiomycosis [22,67]. Further, studies have found that the presence of anti-*Bd* bacteria (including via probiotic treatments; [68]) affects the outcome of exposure to *Bd*. We found that while microbial reduction resulted in a reduction of body condition in the adult life stage (similar to studies on Cuban treefrogs, *Osteopilus septentrionalis*; [6]), the antimicrobial-treated frogs in *Bd*-exposed groups were equally likely to become infected with *Bd* and maintained the same infection intensities over ten weeks of our exposure experiment. Furthermore, we did not observe clinical signs of infection or mortality in any group of frogs. Because the animals were transferred from sterile to non-sterile environments at the time of metamorphosis, we cannot rule out the possibility that the frogs in our study acquired a protective microbiome prior to *Bd* inoculation. The amphibian immune system undergoes transformation during metamorphosis (reviewed in [69]), so this possibility remains to be investigated. Further, absolute microbial abundances were still high for some bacterial phyla in groups treated with antimicrobials, and it is possible this influenced susceptibility in our experiment. Irrespective of microbiome assemblage at the time of metamorphosis, our results corroborate previous reports of *X. laevis* resistance to lethal chytridiomycosis and suggest that further studies on the immune defenses of this species may be warranted.

In addition to future experiments examining susceptibility of *X. laevis* kept under gnotobiotic conditions after metamorphosis, there are several other conditions that could be manipulated in this system. For example, long-term application of antibiotics in Cuban treefrogs had significant effects on tadpole health in terms of survival and time to metamorphosis [6]. In contrast, sub-therapeutic applications of antibiotics are known to enhance growth of animals in agricultural settings [70]. As such, future research could determine if long-term applications of therapeutic concentrations of antimicrobials could have similar effects in *X. laevis* and lead to greater susceptibility of amphibians to *Bd*. Additionally, the mechanisms of low-level antimicrobial treatment on the skin or gut microbiome of amphibians (and the associated disease susceptibility outcomes), and how they may modulate immunity, are not understood. Further testing will be needed to determine how the duration of antimicrobial exposure could alter growth and development, as well as to identify potential developmental windows during which microbial exposure may influence immunological development. Lastly, priority effects (i.e. the sequential order of introduction of different classes of microbes) may affect immunity to specific classes of pathogens (e.g. bacterial versus fungal pathogens [11]), but this possibility remains to be explored in amphibians as well as other model organisms. These potential research directions are intriguing and will likely help us to understand how immunological development can be disrupted or augmented by the microbiome and thereby alter health in later stages of life.

Overall, our results suggest that amphibians may provide an important tool to investigate the influence of the microbiome on the development of the immune system. Because the microbiome can be manipulated with few deleterious effects on body condition, we suggest that further development of this model system will advance our understanding of immune priming in early developmental stages, with potential benefits for expanding our perspectives on the immune equilibrium model and immune system development for a wide range of organisms.

## Data Availability

Data are provided in the electronic supplementary material [71].
